# The IL-15 superagonist ALT-803 enhances anti-CD20 antibody-directed NK cell ADCC and in vivo clearance of B cell lymphomas

**DOI:** 10.1186/2051-1426-2-S3-P168

**Published:** 2014-11-06

**Authors:** Maximillian Rosario, Bai Liu, Lin Kong, Stephanie E Schneider, Emily K Jeng, Peter R Rhode, Hing C Wong, Todd A Fehniger

**Affiliations:** 1 Washington University School of Medicine, St Louis, MO, USA; 2 Altor BioScience Corporation, Miramar, FL, USA

## 

**Figure 1:**
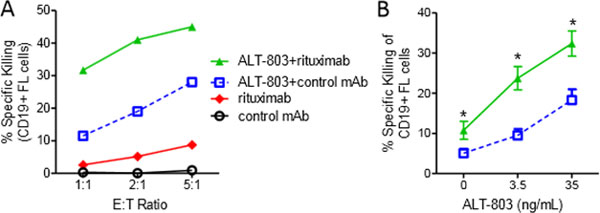
**ALT-803 enhances NK cell ADCC against primary follicular lymphoma**. (A) Representative 4 hour flow-based ADCC assay demonstrating killing of CD19+ lymphoma cells at the indicated effector target ratios after 24 hours of stimulation with 35 ng/ml ALT-803 and 30 minute FL cell labeling with rituximab or control IgG1 monoclonal antibody. Effectors were purified (>95% CD56+CD3-) NK cells. (B) Summary data (N = 5 lymphoma sample targets, N = 15 normal NK cell donors, N = 5 independent experiments) showing the dose-dependent increase in mean ADCC enhanced by ALT-803. *p < 0.001

**Figure 2:**
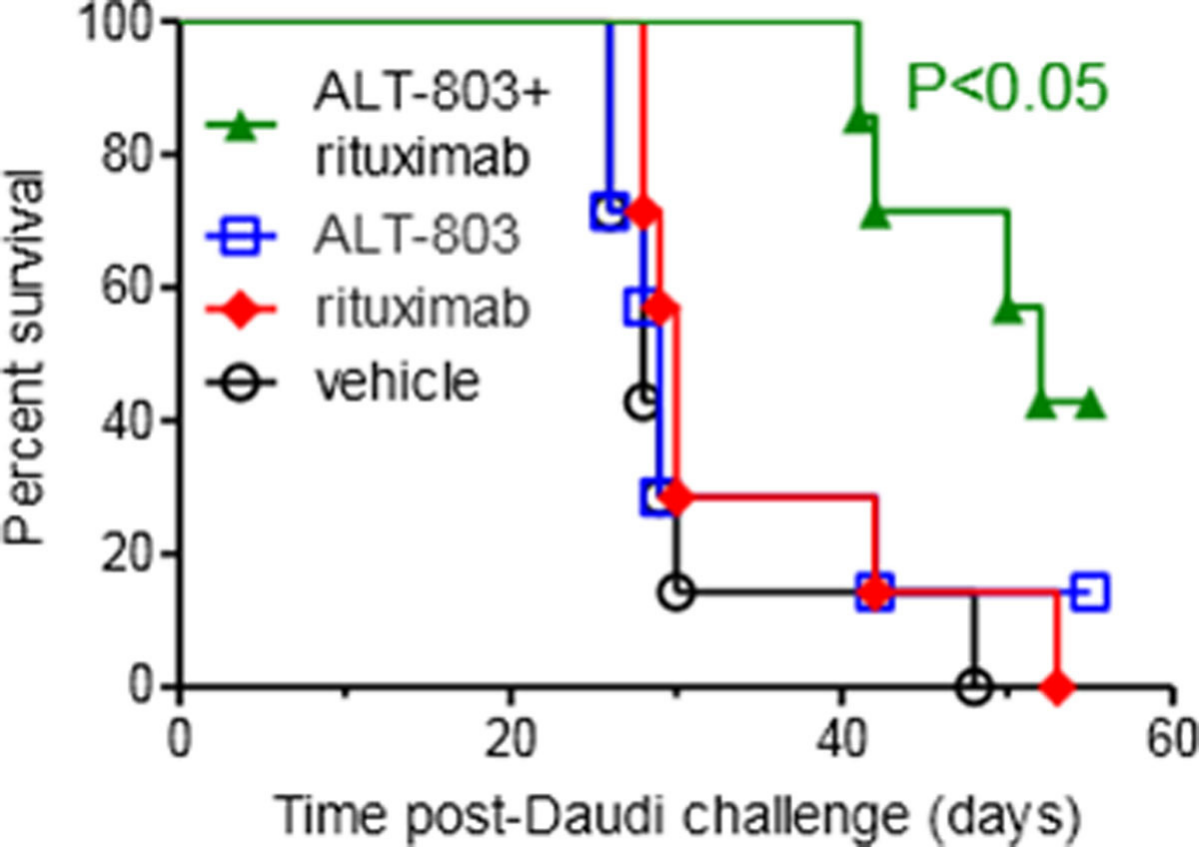
**Alt-803+rituximab protects against lethal Daudi lymphoma challenge**. Kaplan-Meir survival estimates of SCID mice injected iv with 1e7 Daudi lymphoma cells (day 0) and treated with vehicle (PBS). ALT-803 (day 15,18 0.05 mg/kg), rituximab (10 mg/kg day 15), or ALT-803+rituximab (day 15, 18). N = 7 mice per group. Significant (p < 0.05) survival improvement with ALT-803+rituximab compared to vehicle treatment group.

NK cells are innate lymphoid cells that mediate potent anti-tumor responses against B cell malignancies in conjunction with anti-CD20 mAbs. ALT-803 is a superagonist IL-15 and dimeric IL-15Rα-IgG-Fc fusion protein that effectively trans-presents IL-15 and exhibits prolonged in vivo pharmacokinetics compared to rhIL-15. We hypothesized that ALT-803 will augment anti-CD20 mAb (rituximab)-directed NK cell ADCC against B cell lymphomas, facilitating NK cell-mediated lymphoma clearance. Short-term in vitro activation with ALT-803 (0.35-35 ng/mL) increased the expression of the cytotoxic effector protein granzyme B (p < 0.05) in human NK cells. ALT-803 also potentiated rituximab-directed NK cell ADCC against Raji (27% vs. 74%, E:T 25:1, p < 0.01) and Daudi (39% vs. 84%, E:T 2:1, p < 0.05) human B cell lymphoma lines. Moreover, activation of NK cells with ALT-803 significantly augmented CD20-specific ADCC against primary human follicular lymphoma cells in vitro (Figure 1, 11% vs. 33% at a 2.5:1 E:T ratio, p < 0.001). Animal models were employed to assess ALT-803 modulation of NK cell-mediated ADCC against B cell lymphoma in vivo. First, Daudi cells were engrafted into NK cell-competent SCID mice. Groups were treated with vehicle (day 15,18), rituximab (day 15,18), ALT-803 (day 15,18), or rituximab+ALT-803, and assessed for Daudi cell percentages in the BM at day 22. Mice treated with rituximab+ALT-803 combination therapy had significantly reduced Daudi cell burden in BM, compared to rituximab, ALT-803, or vehicle treatment (vehicle vs. ALT-803+rituximab, 38 ± 7% vs. 5 ± 8%, P = 0.005). The enhanced antitumor activity of the combination therapy was ALT-803 dose-dependent (p < 0.02 for 0.02-0.2 mg/kg ALT-803 + rituximab compared to rituximab alone). Furthermore, mice treated with ALT-803+rituximab had superior survival compared to ALT-803 or rituximab monotherapy (Figure 2, P < 0.05). In the second model, Raji B cells expressing luciferase were engrafted into immunodeficient NOD-SCID-γ_c_^-/- ^mice (day 0), and treated with primary human NK cells (day 3) plus vehicle, ALT-803 (0.05 mg/kg q3-4 days), rituximab (day 3), or ALT-803+rituximab. At day 16, ALT-803+rituximab exhibited a significant reduction in Raji signal compared to the control groups (p < 0.05). ALT-803 was well tolerated at all of the administered dose levels in combination with rituximab. Thus, ALT-803 represents an effective IL-15 receptor-agonist that augments NK cell cytotoxic potential and ADCC against malignant B cells in vitro, and significantly increases rituximab-triggered clearance of B cell lymphoma by NK cells in two in vivo models. Based on these findings, a Phase 1/2 clinical trial of ALT-803 plus rituximab is planned for patients with relapsed/refractory indolent NHLs.

